# Mesenchymal Chondrosarcoma in Children and Young Adults: A Single Institution Retrospective Review

**DOI:** 10.1155/2015/608279

**Published:** 2015-06-03

**Authors:** Michael W. Bishop, Jessica M. Somerville, Armita Bahrami, Sue C. Kaste, Rodrigo B. Interiano, Jianrong Wu, Shenghua Mao, Frederick A. Boop, Regan F. Williams, Alberto S. Pappo, Sandeep Samant

**Affiliations:** ^1^Department of Oncology, St. Jude Children's Research Hospital, 262 Danny Thomas Place, Memphis, TN 38105, USA; ^2^Department of Pediatrics, University of Tennessee Health Science Center, Memphis, TN 38105, USA; ^3^Department of Otolaryngology, University of Tennessee Health Science Center, Memphis, TN 38105, USA; ^4^Department of Pathology, St. Jude Children's Research Hospital, 262 Danny Thomas Place, Memphis, TN 38105, USA; ^5^Department of Radiological Sciences, St. Jude Children's Research Hospital, 262 Danny Thomas Place, Memphis, TN 38105, USA; ^6^Department of Radiology, University of Tennessee Health Science Center, Memphis, TN 38105, USA; ^7^Department of Surgery, University of Tennessee Health Science Center, Memphis, TN 38105, USA; ^8^Department of Biostatistics, St. Jude Children's Research Hospital, 262 Danny Thomas Place, Memphis, TN 38105, USA; ^9^Department of Neurosurgery, University of Tennessee Health Science Center, Memphis, TN 38105, USA

## Abstract

*Background*. Mesenchymal chondrosarcoma is an aggressive, uncommon histologic entity arising in bone and soft tissues. We reviewed our institutional experience with this rare diagnosis. *Methods*. We conducted a retrospective chart review on patients with mesenchymal chondrosarcoma over a 24-year period. Clinicopathologic and radiographic features were reviewed. *Results*. Twelve patients were identified. Nine were females; median age was 14.5 years (1.2–19.7 years). The most common site was the head/neck (7/12). Disease was localized in 11/12 patients (one with lung nodules). Six with available tissue demonstrated *NCOA2* rearrangement by FISH. Six underwent upfront surgical resection, and six received neoadjuvant therapy (2 chemotherapy alone and 4 chemotherapy and radiation). All patients received adjuvant chemotherapy (most commonly ifosfamide/doxorubicin) and/or radiation (median dose 59.4 Gy). At a median follow-up of 4.8 years, 5-year disease-free survival and overall survival were 68.2% (95% CI 39.8%, 96.6%) and 88.9% (95% CI 66.9%, 100%). Two patients had distant recurrences at 15 and 42 months, respectively. *Conclusion*. Aggressive surgical resection of mesenchymal chondrosarcoma with chemoradiotherapy yields excellent local control and may reduce likelihood of late recurrence. Characterization of downstream targets of the *HEY1-NCOA2* fusion protein, xenograft models, and drug screening are needed to identify novel therapeutic strategies.

## 1. Introduction

Mesenchymal chondrosarcoma comprises 2–10% of all chondrosarcomas [1–5]. This histological subtype occurs in both osseous and extraosseous tissues [6] and has a tendency for late local and disseminated recurrence [1, 2, 4, 7]. In children and adolescents, mesenchymal chondrosarcoma accounts for up to 25% of all chondrosarcomas [7]. The rarity of this histologic entity has made it difficult to analyze the natural history and best therapeutic options for these patients [8–10]. Thus, we retrospectively reviewed our institutional experience with pediatric and adolescent patients diagnosed with mesenchymal chondrosarcoma over the past 24 years.

## 2. Materials and Methods

Following Institutional Review Board approval, we conducted a retrospective chart review on patients presenting with the diagnosis of mesenchymal chondrosarcoma to St. Jude Children's Research Hospital from January 1, 1990, to May 30, 2014. Abstracted data included clinical features, outcome, radiographs, and therapy received including chemotherapy, radiation, and surgery. Available radiographic imaging was reviewed by one of the authors (Sue C. Kaste).

Radiographic response to preoperative therapy was determined using the revised RECIST (response evaluation criteria in solid tumors) guideline version 1.1 [11]. Patients were considered to have partial response with at least a 30% decrease in the largest diameter of the primary tumor between imaging obtained at the onset of treatment and preoperative imaging, progression of disease with a 20% or greater increase in tumor volume between imaging studies, and stable disease with neither sufficient decrease to qualify as partial response nor increase to qualify as progressive disease. An elliptical volumetric model with volume equivalent to 0.5 times the product of the three largest perpendicular diameters was also evaluated, with partial response of 64% of greater decrease in tumor volume and progression of disease of 40% increase in volume [12]. Pathology specimens were reviewed by one of the authors (Armita Bahrami). Fluorescence in situ hybridization (FISH) was performed to evaluate* NCOA2* rearrangement in samples when tissue was available.

Statistics were calculated using SAS v9.3 (SAS Institute Inc., Cary, NC). Overall survival (OS) and disease-free survival (DFS) were calculated using Kaplan-Meier models as previously published [13]. OS was calculated using time from diagnosis to death (due to all causes) or to last follow-up. DFS was calculated as time from diagnosis to recurrence or progression of disease or death. Patients who had not met criteria for an event were censored at the time of last follow-up. Log-rank analysis was used to assess association of variables with OS and DFS.

## 3. Results

### 3.1. Clinical Characteristics

Twelve patients with mesenchymal chondrosarcoma were identified. The clinical characteristics, treatment, and outcomes are displayed in Table 1. Nine patients were females; median age at diagnosis was 14.5 years (range: 1.2–19.7 years). Seven patients presented with disease arising in the head and neck region, most commonly involving the orbit (2). Other involved sites included the chest wall (3), intra-abdominal and lumbar paraspinal disease. Five patients presented with tumor arising from bony structures. Eleven patients presented with localized disease, and one presented with metastases to the lung parenchyma. Clinical symptoms at diagnosis were characteristics of mass lesions arising in the involved compartment, including pain, swelling/distention, and proptosis; the two patients with intraspinal disease presented with neurologic deficits including extremity weakness and incontinence. Radiographic features of the lesions included a soft tissue mass with calcifications present throughout, bony destruction of primary/adjacent osseous structures, and variable patterns of postcontrast enhancement; internal septations were visualized in two patients. On MRI, lesions were typically isointense to muscle and had decreased signal intensity compared to fat on T1-weighted imaging. Increased intensity compared to muscle was seen on T2-weighted imaging. Tumor margins were well defined with smooth margins and occasional lobulations. Six patients had tissue available for FISH analysis; all six were found to have rearrangement of* NCOA2*.

### 3.2. Treatment and Outcomes

All patients were evaluable for treatment and response to therapy. Six patients underwent upfront surgical resection of disease. Of these, one had a complete surgical resection with negative margins, three had microscopic residual disease following surgical intervention, and one patient had gross residual tumor. One patient with chest wall mass and pulmonary metastases at presentation had gross total resection of primary tumor with negative margins but was unable to achieve surgical clearance of metastatic lung nodules.

Six patients with localized disease whose tumors were not felt to be resectable at diagnosis due to size or location received neoadjuvant therapy (Table 1). Chemotherapy alone was administered upfront in two patients, including a male with rapidly progressive nasal cavity primary tumor (Patient 11) who received a single course of chemotherapy to stabilize disease prior to surgery and was treated postoperatively with radiation alone. Four patients received both chemotherapy and radiation prior to surgery. Patient 6 received 50 Gy of external beam radiation after demonstrating no response to initial chemotherapy cycles and received an additional 20 Gy for postoperative consolidation. Three patients received combination chemoradiotherapy as per a multicenter prospective clinical trial for nonrhabdomyosarcoma soft tissue sarcomas [14]. Responses to neoadjuvant therapy are shown in Table 1; one patient demonstrated progressive disease with 42% increase in size of the lesion by RECIST criteria but was considered stable by elliptical volumetric modeling (23% increase). Another patient was stable by RECIST criteria with 25% decrease in largest diameter but was defined as a partial responder by volumetric modeling with 68% decrease in residual tumor volume. The other four patients had stable disease by both methods of evaluation. Following neoadjuvant treatment, four patients were able to have their tumor completely resected with negative margins; all are alive with no evidence of disease at a median of 6.5 years from diagnosis (range 5.2–10.7 years). One of the two patients with microscopic residual disease developed disseminated bony recurrence 42 months from diagnosis and has received several second-line therapies.

All patients received adjuvant therapy following surgical resection. Overall, eight patients received adjuvant chemotherapy; the most common regimen consisted of ifosfamide and doxorubicin. Adjuvant chemotherapy was administered for most patients who presented with large primary tumors (>5 cm) or with unresectable disease at diagnosis. Radiation was administered postoperatively to nine patients; the median cumulative dose of radiation received was 59.4 Gy (range 45–70 Gy). The majority of patients receiving adjuvant radiotherapy had residual microscopic (5) or macroscopic (1) disease; two patients with tumors greater than 10 cm at diagnosis received definitive radiation in addition to postoperative chemotherapy. Two patients who had microscopic margins after surgical resection received definitive radiation treatment without concomitant chemotherapy. Additional two patients who had small localized tumors that were completely resected were treated with adjuvant chemotherapy without further radiation (both had received neoadjuvant chemoradiotherapy). The sole patient with metastatic disease at diagnosis was treated with ifosfamide and doxorubicin after resection of his primary chest wall mass and demonstrated a decrease in the number of pulmonary nodules. Despite thoracotomies to remove residual disease, he rapidly developed worsening metastatic progression and died of disease 15 months after diagnosis.

**Figure 1:**
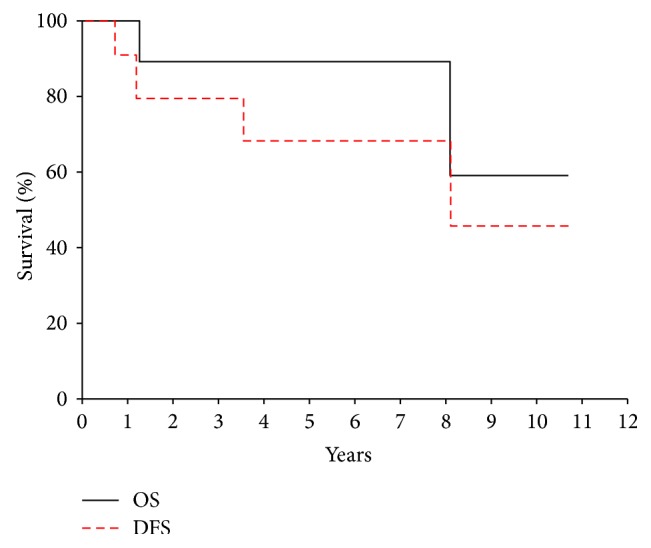
Overall survival (OS) and disease-free survival (DFS) for all patients with mesenchymal chondrosarcoma.

**Figure 2:**
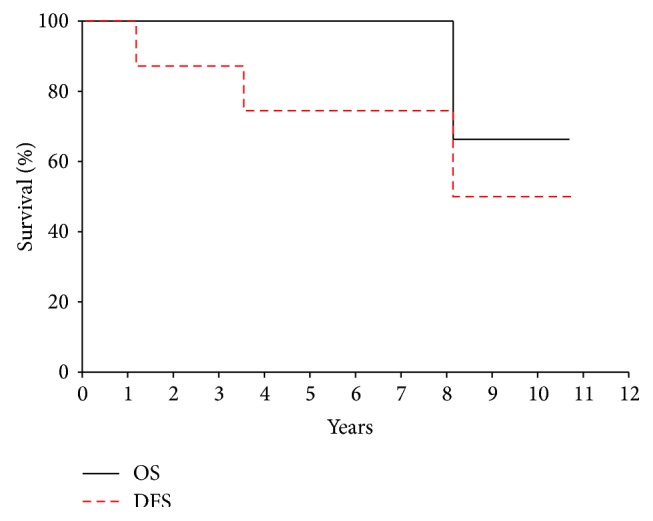
Overall survival (OS) and disease-free survival (DFS) for patients with mesenchymal chondrosarcoma presenting with localized disease at diagnosis.

At a median follow-up time of 4.8 years, the 5-year disease-free survival (DFS) and overall survival (OS) for all patients were 68.2% (95% CI 39.8%, 96.6%) and 88.9% (95% CI 66.9%, 100%), respectively (Figure 1). 5-year DFS and OS for patients with localized disease at diagnosis were 75% (95% CI 47.2%, 100%) and 100% (Figure 2). None of the patients have developed a local recurrence. Sites of distant recurrence in two patients included the lungs and multifocal bony sites. A third patient developed therapy-related myelodysplastic syndrome and acute myeloid leukemia (MDS/AML), received hematopoietic stem cell transplantation, and currently has no evidence of disease. Despite the small number of patients available for review, log-rank analysis demonstrated that the ability to achieve gross total resection, regardless of margin status, was necessary for prolonged OS (*P* = 0.02) but was not significant for DFS. Other factors including sex, tumor size, head/neck location, osseous/extraosseous location, and type of neoadjuvant treatment did not correlate with DFS or OS.

## 4. Discussion

Our report summarizes the clinical, pathological, and molecular characteristics of 12 patients with mesenchymal chondrosarcoma who were seen at our institution over a 24-year period, confirming the rarity of this histologic entity. In adults, mesenchymal chondrosarcoma accounts for 2 to 10% of all chondrosarcomas, with an estimated number of 215 cases per year in the United States [5]. In children and adolescents, mesenchymal chondrosarcoma accounted for 0.2% (15 of 7000) of all bone and soft tissue sarcomas enrolled on German cooperative soft tissue sarcoma and osteosarcoma trials [15]. A recent Children's Oncology Group study for nonrhabdomyosarcoma soft tissue sarcomas registered 551 eligible patients; only 5 mesenchymal chondrosarcomas (0.9%) were identified [14]. The exceptionally low incidence of this disease has made study of its clinical behavior extremely difficult.

Chemotherapy for most patients in our cohort consisted of a dose-intense regimen of ifosfamide and doxorubicin, agents which have historically demonstrated activity in adult and pediatric soft tissue sarcomas [16]. Prior single institutional studies have used Ewing sarcoma-based therapeutic regimens for treatment of mesenchymal chondrosarcoma [15, 17]; survival outcomes for our cohort are relatively similar. Our study suggests that mesenchymal chondrosarcomas are relatively chemoresistant since only one of six evaluable patients achieved a partial response to therapy by volumetric modeling. This is similar to adult data demonstrating only 31% objective response to chemotherapy [18]; in the CWS/COSS study, only two of seven patients evaluable for response demonstrated reduction greater than 66% using a tumor volume model. The role of radiation therapy for mesenchymal chondrosarcoma has been questioned, with conflicting results in the literature [2, 3, 10, 19]. However, aggressive surgical resection in combination with chemotherapy and radiation was successful in achieving durable local remission within our cohort, with no local recurrences. Four patients who received neoadjuvant treatment, complete surgical resection of disease, and adjuvant therapy are all alive with no evidence of disease at 5.3 to 10.7 years from diagnosis, suggesting a benefit to combined modalities for treatment in reducing the likelihood of late distant recurrence. Other studies in patients with unresected nonrhabdomyosarcoma soft tissue sarcomas have demonstrated that a combined approach that includes local control measures is critical for survival in these patients [20, 21]. Close monitoring for cardiac and renal events as well as secondary malignancies and disease recurrence remain vital for the long-term management of these patients, as some studies of mesenchymal chondrosarcoma have reported 10- year survival rates of 37% or less [7, 17, 19].

Our study also highlights the importance of* HEY1-NCOA2* rearrangement in mesenchymal chondrosarcoma and its role as a diagnostic marker but also its potential for therapeutic advances [22, 23].* HEY1* is a downstream effector of the Notch signaling pathway [22, 24]; aberrant signaling of the Notch-Hey1 axis has been demonstrated to impede cell differentiation and promote proliferative capabilities in embryonal rhabdomyosarcoma cell lines [25].* NCOA2* mutations have been identified in adult melanomas and lung cancer, and amplification of the* NCOA2* locus may act as an oncogene in prostate cancer via upregulation of androgen receptor transcriptional output [26]. In pediatrics,* NCOA2* rearrangements have been identified in acute leukemia and rhabdomyosarcoma [27–29]. Further characterization of* Notch/HEY1* target gene expression and* NCOA2*-related nuclear receptor target genes in mesenchymal chondrosarcoma may provide insight into avenues for treatment with targeted therapies.

The rarity of mesenchymal chondrosarcoma and other pediatric soft tissue sarcomas requires a uniform and collaborative effort to better study these diseases. Recently, a multicenter prospective clinical trial for nonrhabdomyosarcoma soft tissue sarcomas (COG ARST0332) was completed, and a successor trial (ARST1321) is now activated at many pediatric centers. Given the low incidence of unique histologic variants such as mesenchymal chondrosarcoma, these clinical trials will be critical for obtaining tissue to develop cell lines and patient-derived xenografts for the purposes of genomic analyses, functional studies, and drug screening. This model has been successful for development of therapies for other solid malignancies such as retinoblastoma [30], and its implementation in concert with a large prospective trial would yield new opportunities for therapeutic strategies.

Our study has several limitations, including the small number of patients given the rarity of diagnosis and the retrospective nature of the analysis. Because of the prolonged clinical course previously observed in this entity, the median follow-up time of 4.8 years is inadequate to evaluate for late, distant recurrences. Despite these limitations, our study comprises one of the largest exclusively pediatric cohorts of mesenchymal chondrosarcoma reported to date. Because some prior reports have suggested limitations of RECIST criteria for pediatric solid tumors [31], this analysis incorporated measures of response by both RECIST criteria and elliptical tumor volumetric modeling. Volumetric models were used to define response in previous analyses of mesenchymal chondrosarcoma [15] and are currently being evaluated in several contemporary studies including the recently closed COG study for nonrhabdomyosarcoma soft tissue sarcomas [14].

In summary, we have identified a cohort of pediatric patients with mesenchymal chondrosarcoma who received chemotherapy and/or radiation in addition to local control. Five-year overall survival curves suggest that current treatment modalities allow for excellent local control of disease, but further longitudinal observation of our cohort will be necessary to determine whether aggressive local therapy in combination with systemic chemotherapy will yield durable survival outcomes. International collaboration is essential to further progress for the treatment of this rare entity.

## Figures and Tables

**Table 1:** Characteristics, treatment, and outcome of children and adolescents with mesenchymal chondrosarcoma.

Patient	Age (yr)	Sex	Primary site	Size (cm)	Osseous versus extraosseous	Neoadjuvant therapy	Response (RECIST)	Response (volumetric)	Extent of surgery	Adjuvant therapy	Outcome (yr)
1	15.2	F	Abdominal mesentery	12.7	Extraosseous	—	—		CR	RT (55.2 Gy) CH (I, D)	NED (0.7)
2	14.5	F	Chest wall	2.5	Osseous	—	—		+margin	Brachytherapy (15 Gy) RT (45 Gy)	NED (8.7)
3	19.7	F	Pterygopalatine fossa	5.3	Extraosseous	—	—		+margin	RT (59.4 Gy)	AWD (1.3)
4	13.9	F	Maxillary sinus	5.3	Osseous	—	—		+margin	CH (I, D) RT (55.8 Gy)	NED (0.3)
5	1.3	F	Paraspinal	6.0	Extraosseous	—	—		Gross residual disease	RT (60.7 Gy) CH (I, C)	Died (8.1), renal failure
6	17.3	F	Maxilla	5.5	Osseous	CH (I, D, V) RT (50 Gy)	SD	SD	CR	RT (20 Gy)	NED (10.7)
7	11.7	M	Chest wall	10.4	Osseous	CH (I, D, V, E)	SD	SD	CR	CH (I, D, V, E) RT (55.8 Gy)	NED (5.3)
8	9.8	F	Orbit	2.5	Extraosseous	CH (I, D) RT (45 Gy)	SD	SD	CR	CH (I, D)	NED (6.8)
9	17.4	F	Orbit	2.1	Extraosseous	CH (I, D) RT (45 Gy)	SD	PR	CR	CH (I, D)	NED (6.1)
10	12.8	F	Intraspinal	3.1	Extraosseous	CH (I, D) RT (45 Gy)	PD	SD	+margin	CH (I, D) RT (10.8 Gy)	AWD (4.3)
11	12.1	M	Nasal cavity	6.5	Extraosseous	CH (I, D)^*∗*^	SD	SD	+margin	RT (59.4 Gy)	NED (1.2)
12	16.5	M	Chest wall	2.5	Osseous	—	—		Primary resected^*∗∗*^	CH (I, D)	DOD (1.3)

^*∗*^Patient received one cycle of neoadjuvant chemotherapy only; ^*∗∗*^Primary tumor grossly resected, unable to be cleared of metastatic lung nodules. RT: radiotherapy; Gy: Gray; CH: chemotherapy; I: ifosfamide; D: doxorubicin; C: carboplatin; V: vincristine; E: etoposide; SD: stable disease; PR: partial response; CR: complete resection; NED: no evidence of disease; AWD: alive with disease; DOD: died of disease.
